# A large undifferentiated sarcoma of the liver in a 13-year-old girl treated with anatomical resection: a case report and review of the literature

**DOI:** 10.1186/s12876-021-02076-x

**Published:** 2022-01-03

**Authors:** Ali Bahador, Mehdi Forooghi, Reza Shahriarirad, Bita Geramizadeh, Maryam Ataollahi, Hooman Kamran

**Affiliations:** 1 Department of Pediatric Surgery, Nemazee Hospital, Shiraz University of Medical Sciences, Shiraz, Iran; 2 Student Research Committee, Shiraz University of Medical Sciences, Shiraz, Iran; 3 Thoracic and Vascular Surgery Research Center, Shiraz University of Medical Sciences, Shiraz, Iran; 4 Shiraz Transplant Research Center (STRC), Shiraz University of Medical Sciences, Shiraz, Iran; 5 Department of Pathology, Shiraz University of Medical Sciences, Shiraz, Iran; 6 Gastroenterohepatology Research Center, Shiraz University of Medical Sciences, Shiraz, Iran; 7 Nemazee Hospital, Zand Street, Nemazee Square, 71936-13311 Shiraz, Iran

**Keywords:** Undifferentiated embryonal sarcoma, UESL, Hepatectomy, Liver resection, Malignant liver tumor

## Abstract

**Background:**

Undifferentiated embryonal sarcoma of the liver (UESL) is a rare liver tumor accounting for 6–13% of primary liver tumors. Accurate preoperative diagnosis is difficult, with a rather high misdiagnosis rate. Herein, we reported a very large UESL treated with anatomical resection. Our case is amongst the largest pediatric UESLs in the literature.

**Case presentation:**

Herein, we report a 13-year-old girl presenting with right upper quadrant abdominal pain, postprandial vomiting, and abdominal distention, in which radiographic imaging demonstrated a huge UESL (28 × 20 × 12 cm). The patient was treated with partial hepatectomy and the 5 kg tumor was removed. The patient was discharged in good condition, with no significant complaints in her follow-up.

**Conclusions:**

Although different treatment strategies have been reported for UESL cases, anatomical resection is still the main treatment approach, especially for large tumors.

## Background

Undifferentiated embryonal sarcoma of the liver (UESL) is a rare liver tumor accounting for 6–13% of primary liver tumors [1]. It is also termed as malignant mesenchymoma because of its mesenchymal origin [2]. Commonly, UESL is seen in children with a peak between 6 and 10 years but has also been reported in adults [3]. Common clinical characteristics of the disease include abdominal pain, palpable mass, and fever [4], while radiological features include cystic lesions in abdominal computed tomography (CT) or magnetic resonance imaging (MRI) [5]. However, because of the low specificity of imaging, lab findings, signs, and symptoms, it can be misdiagnosed as other liver lesions [6]. Although UESL is considered a poor prognostic tumor, combined surgical excision and chemotherapy are considered a favorable treatment option, especially in large-sized tumors [1].

Herein, we intend to present one of the largest pediatric UESLs reported in the literature with 5 kg weight and 28 × 20 × 12 cm size.

## Case presentation

A 13-year-old girl with no significant past medical history presented with abdominal pain in the right upper quadrant (since three months ago), postprandial vomiting, and abdominal distention (since two months ago). A local physician had visited her, and abdominal sonography and CT scan had been requested. Abdominal sonography had shown heterogeneous predominantly solid mass (14 × 14 cm) with cystic changes located in the upper posterior part of the right lobe of the liver. Also, in the CT scan of the abdomen, a well-defined soft tissue density mass (12 × 11 × 9 cm) in the right lobe of the liver had been reported. She was referred to our hospital for further evaluation.

In physical examination, she was afebrile with normal vital signs and no signs of jaundice; however, abdominal distention and a palpable mass in the right upper quadrant with severe tenderness were distinct. Laboratory data showed leukocytosis and elevated liver enzymes and direct hyperbilirubinemia. Alpha-fetoprotein was within the normal range. The cystic lesion had been interpreted as a benign lesion of the liver.

**Fig. 1:**
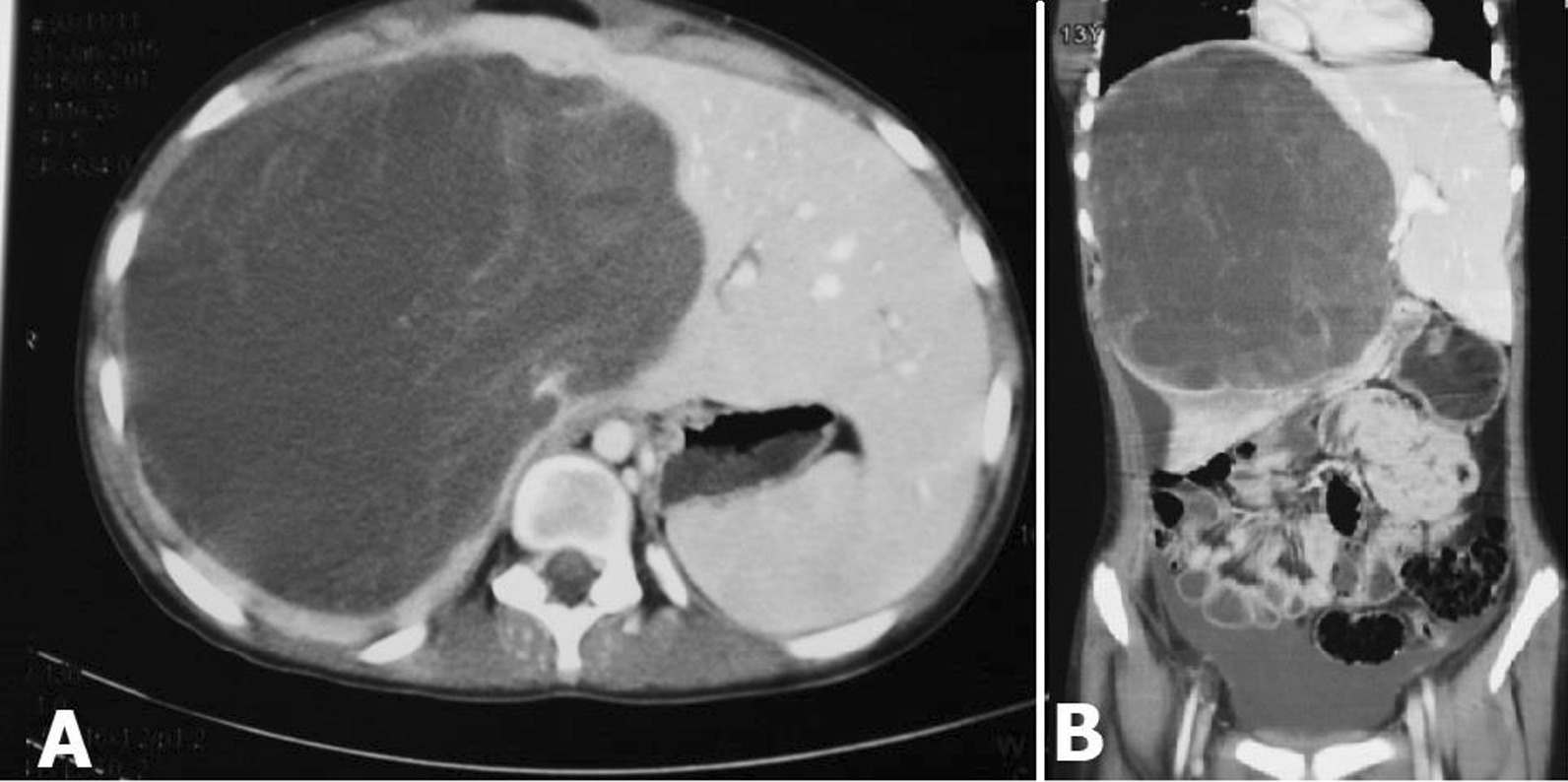
Computed tomography images of a hypodense lesion in the right lobe of the liver, in favor of a huge undifferentiated embryonal sarcoma of the liver

An abdominal CT scan revealed a large hypodense lesion (200 × 156 × 172 mm) occupying the right lobe of the liver. Faint peripheral and internal septation was seen in the mass. Mild to moderate ascites was reported in the abdomen and pelvic cavity. Also, the gallbladder was normal in configuration with no stone or wall thickening (Fig. 1). The possibility of a large mesenchymal hamartoma or an atypical hemangioma was considered. Therefore, the patient was scheduled for operation without receiving any episodes of radiotherapy or neoadjuvant chemotherapy.

**Fig. 2:**
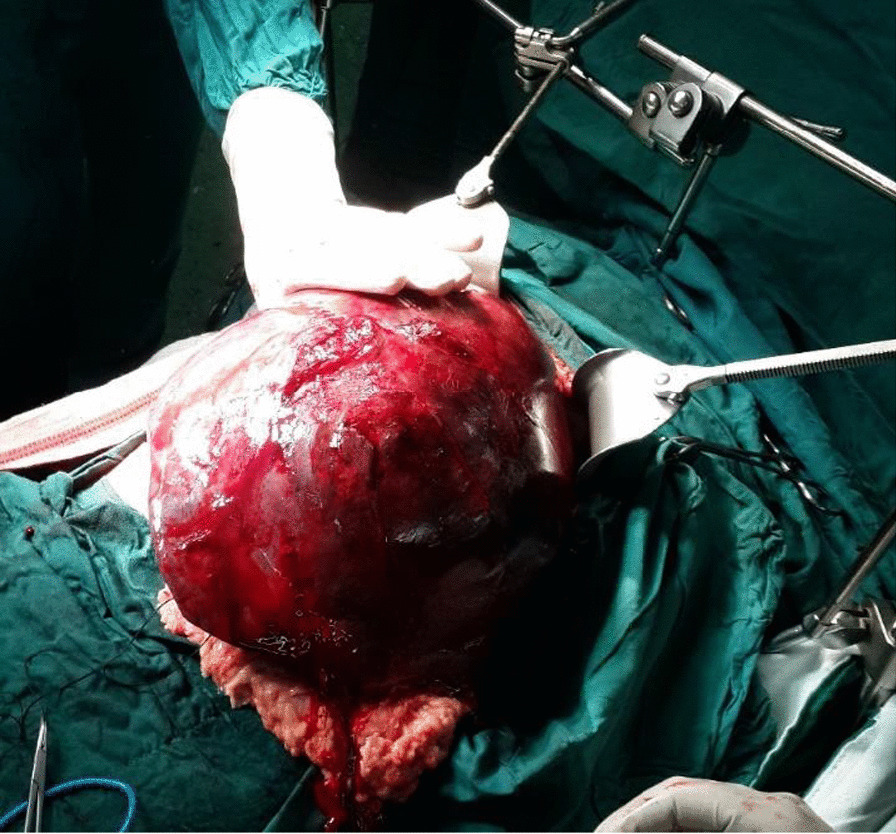
Surgical resection of a huge embryonal sarcoma of the liver

**Fig. 3:**
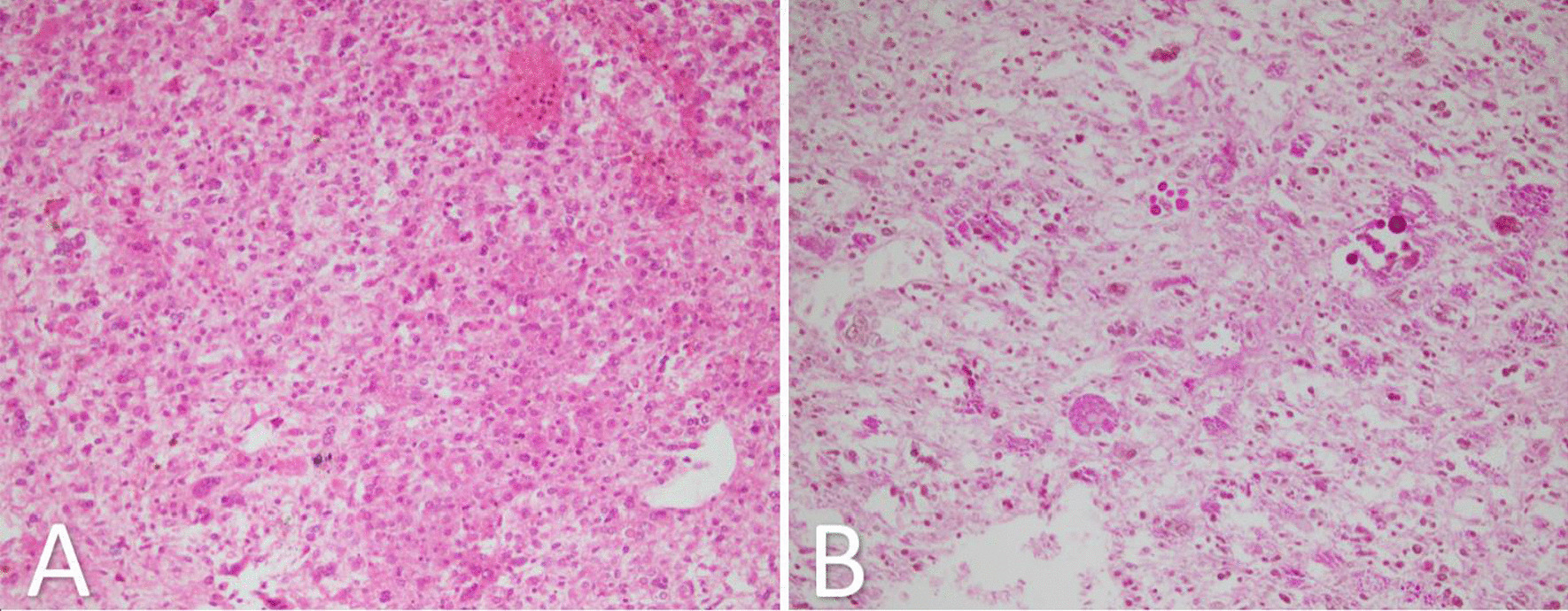
**A** A section from the embryonal sarcoma of the liver which shows highly cellular tumor with pleomorphic cells in myxoid and necrotic background (H&E ×250); **B** periodic acid-Schiff stain after diastase (dPAS) showing intracellular PAS positive diastase resistant hyaline globules

The patient underwent exploratory laparotomy. Intraoperative findings showed a huge mass in the right liver lobe (5 kg) (Fig. 2); therefore, right hepatectomy was carried. Pathologic examination demonstrated a large multilobulated mass in the liver (28 × 20 × 12 cm), with extensive hemorrhage and necrosis. The mass was hemorrhagic, solid, and cystic with a gray to dark red color involving hilum. Microscopic examination of the tumor showed highly pleomorphic cells with many multinucleated forms in a highly necrotic background. Tumor cells showed hyperchromatic, highly anaplastic nuclei with many mitoses (Ki-67 proliferative index was also high, around 40%). PAS with diastase stain showed intra-cytoplasmic PAS positive and diastase resistant globules (Fig. 3). Tumor cells were reactive with vimentin but non-reactive with smooth muscle antigen (SMA), desmin, CD34, cytokeratin, hepatocyte-specific antigen (HepPar-1), Myo-D1, and S100. Lymphovascular invasion and satellite nodules were absent, and no lymph node was identified. Negative epithelial marker (cytokeratin) excluded epithelial tumors such as hepatoblastoma and hepatocellular carcinoma. Another important differential diagnosis was embryonal rhabdomyosarcoma, which was also excluded by negative desmin and Myo-D1.

**Fig. 4:**
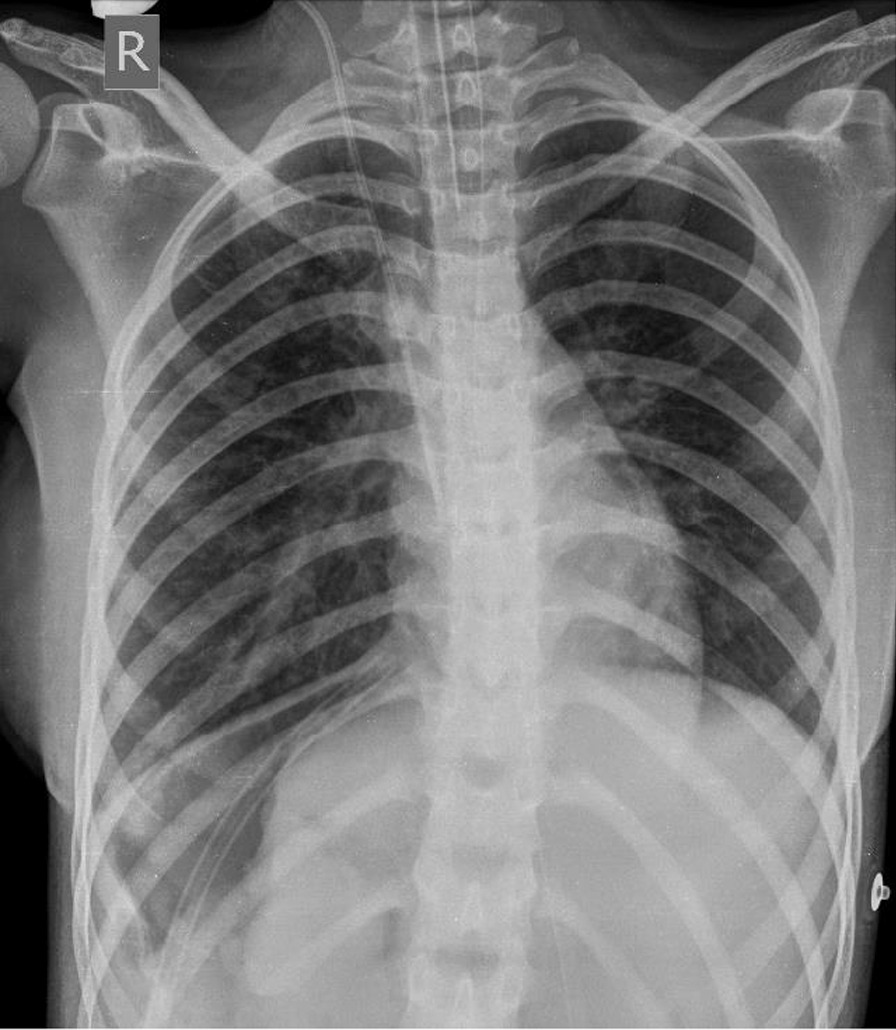
Chest X-ray after surgical resection of a huge undifferentiated embryonal sarcoma of the liver

She was tumor-free following the surgery and discharged 8 days postoperatively in good condition (Fig. 4). She was also prescribed an adjuvant chemotherapy regimen, which during her follow-up, we discovered that she refused to receive. The patient has been symptom-free for 6 years after surgery, with no evidence of recurrence of the tumor.

## Discussion and conclusions

UESL is a mesenchymal-derived rare liver tumor that is more common in children. Because of its rarity and lack of specificity in clinical presentations and similar imaging to other cystic lesions, it can be misdiagnosed before surgery. Early diagnosis is important because of the tumor's poor prognosis, and even a survival time of less than a year has been reported by Stocker and Ishak in 1978 [7]. However, with the improvements in treatment approaches in the last decades, UESL survival time has been improved. Besides, complications can occur in some cases with UESL. UESL cases have been reported as rare entities in the literature. Wu et al. reported a total of 308 UESL cases, including their case, in 131 articles till 2019 [4]. In the mentioned study, 219 (71.1%) of the cases were children (under 18 years). Herein, we reported a 13-year-old girl with a very large UESL treated only with anatomical resection. Table 1 demonstrates three cases of pediatric UESL, along with our case, with tumor sizes of above 20 cm [8–10].

**Table 1:** Cases of undifferentiated embryonal sarcoma of the liver with large-sized tumors

References	Age (year), Sex	Tumor size/weight	Treatment	Follow-up
Baron et al. [8]	13, male	25 cm, 3 kg	Right hepatic trisegmentectomy, postoperative chemotherapy (six cycles: cisplatin and adriamycin with vincristine, ifosfamide, and etoposide)	48 months tumor-free following the treatment
Kamrani et al. [9]	11, female	25 × 19 × 14 cm (on T2-weighted MRI)	Left lateral hepatectomy	(not mentioned)
Plumblee et al. [10]	8, male	21 × 14 cm (the largest size observed in imaging)	Neoadjuvant chemotherapy (one cycle: vincristine, actinomycin D, cyclophosphamide; three cycles: ifosfamide/doxorubicin), orthotopic liver transplant, adjuvant chemotherapy (two cycles: ifosfamide/doxorubicin; one cycle: ifosfamide)	Well at 18 months post-transplant
Bahador et al. (our case)	13, female	28 × 20 × 12 cm (weighing 5 kg)	Exploratory laparotomy followed by right hepatectomy	6 years tumor-free

*MRI* magnetic resonance imaging

Different treatment strategies have been used in the reported UESLs. Total or partial resection of the liver is the main treatment approach. Also, chemotherapy can be beneficial. The combination of surgical resection and chemotherapy is one of the best approaches for UESL [1]. However, a standard chemotherapy regimen is not considered for UESL. Although surgical resection is the main treatment strategy for UESL, in some conditions such as large tumor size, attachment to vital structures, and no response to neoadjuvant chemotherapy, it may become contraindicated. In these conditions, orthotopic liver transplantation could be a beneficial approach [10]. However, it is reported that extrahepatic tumor spread cannot be considered as a contraindication for surgical resection [4]. Despite the mentioned conditions, we showed that even a very large-sized UESL tumor with the size of 28 × 20 × 12 cm in a pediatric could be treated only with surgical resection and without neoadjuvant and/or adjuvant chemotherapy. Even though we referred the patient for completing her treatment with adjuvant chemotherapy, considering the very large size of the tumor, she refused chemotherapy. However, the patient reported no signs of recurrence in the follow-up six years following the surgery.

In addition to the mentioned treatments, other options have been utilized in some cases. Radiation therapy is used in several cases, but its effectiveness is not completely determined [11]. Also, orthotopic liver transplantation has been an effective alternative to total surgical resection [10, 12].

Some complications can occur in UESL cases, such as extrahepatic metastasis, which has been observed in organs such as the lung, diaphragm, and peritoneum, with a prevalence of approximately 20% [4]. Direct involvement of the heart with inferior vena cava tumor extension to the right atria has been reported [13]. Also, tumor spontaneous rupture, which is a life-threatening complication, should be considered [14, 15]. Furthermore, UESL recurrence rate is high [16]; so, an early diagnosis is necessary to prevent fatal outcomes.

Finally, UESL can be misdiagnosed with other hepatic lesions. Imaging shows a cystic lesion, and clinical manifestation and laboratory tests are not specific. A multidisciplinary approach is needed for an accurate preoperative diagnosis; however, a lack of understanding of UESL makes it difficult to diagnose the tumor initially [6]. The differential diagnosis of UESL in children can be hepatoblastoma, mesenchymal hamartoma, and embryonal rhabdomyosarcoma of the biliary tree (Table 2) [3, 17–24]. Besides, although immunohistochemistry is a helpful test for accurate diagnosis of the tumor and ruling out other liver tumors, UESL has no specific markers [25].

**Table 2:** Comparison of undifferentiated embryonal sarcoma of the liver, hepatoblastoma, mesenchymal hamartoma and embryonal rhabdomyosarcoma

Type of tumor	Radiological appearance	Clinical manifestations	Pathology	Immunohistochemistry	Location
UESL	Solid (USG), cystic mass with internal septations and serpiginous vessels (CT), hypointense (T1-weighted MRI), hyperintense (T2 MRI)	Abdominal pain, palpable mass, fever	Spindle and stellate pleomorphic cells in a myxoid stroma	Vimentin, *α*1-antitrypsin, CD10, CD56, CD68, BCL2	Solitary mass in the right lobe of the liver
Hepatoblastoma	Sometimes with lesion and hemorrhage in the mass (USG), attenuation values Between water and normal liver parenchyma (CT), bands with low signal intensity compared with liver (T1-weighted MRI), bands with high signal intensity compared with liver (T2-weighted MRI)	Abdominal distension, abdominal mass	Various histological subtypes in different stages of liver development	CK19, beta-catenin, epcam	Mostly, a solitary mass in the right lobe of the liver
Mesenchymal hamartoma	Cystic mass with internal septations (USG), cystic mass with septal and solid stromal enhancement (CT/MRI), variable intensity (T1-weighted MRI), high signal intensity (T2-weighted MRI)	Abdominal distension, abdominal mass	Lobular growth of myxomatous connective tissue with branching bile ducts	Vimentin, *α*1-antitrypsin, actin, cytokeratin, desmin	Usually, a large solitary mass in the right lobe of the liver
Embryonal rhabdomyosarcoma	Dilation of the intra- and extrahepatic bile ducts with an intraductal mass (USG), signal of skeletal muscle (T1-weighted MRI), hyperintense (T2-weighted MRI)	Jaundice, abdominal pain, fever, vomiting	A condensed layer of neoplastic subepithelial cellularity (cambium layer)	Desmin, myogenin, Myo-D1	Biliary tract

*UESL* undifferentiated embryonal sarcoma of the liver, *USG* ultrasonography, *CT* computed tomography, *MRI* magnetic resonance imaging

In conclusion, although the combination of surgical resection and chemotherapy is the optimal treatment for UESL, we showed that treatment of very large tumors, such as in our case, is possible only with anatomical resection. However, disposing of the need for adjuvant chemotherapy still requires further evidence and studies. Generally, there are two important steps in the management and treatment of UESL:

1. An early diagnosis to prevent the possible complications and increase the chance of survival in the patients; keeping in mind the differential diagnosis of the tumor
2. Considering therapeutic options and choosing the best treatment for the patient, depending on the patient’s condition; so far, surgical excision plays the main role in the treatment.

## Data Availability

Data of the patient can be requested from authors. Please write to the corresponding author if you are interested in such data.

## Abbreviations

UESL: Undifferentiated embryonal sarcoma of the liver
CT: Computed tomography
MRI: Magnetic resonance imaging
PAS: Periodic acid-Schiff

## References

[1] Murawski M, Scheer M, Leuschner I, Stefanowicz J, Bonar J, Dembowska-Bagińska B (2020). Undifferentiated sarcoma of the liver: multicenter international experience of the Cooperative Soft-Tissue Sarcoma Group and Polish Paediatric Solid Tumor Group. Pediatric Blood Cancer.

[2] Stout AP (1948). Mesenchymoma, the mixed tumor of mesenchymal derivatives. Ann Surg.

[3] Martins-Filho SN, Putra J (2020). Hepatic mesenchymal hamartoma and undifferentiated embryonal sarcoma of the liver: a pathologic review. Hepat Oncol.

[4] Wu Z, Wei Y, Cai Z, Zhou Y (2020). Long-term survival outcomes of undifferentiated embryonal sarcoma of the liver: a pooled analysis of 308 patients. ANZ J Surg.

[5] Thombare P, Verma M, Shah K, Doshi H, Verma G, Patkar D (2020). Undifferentiated embryonal sarcoma of liver: paradoxical imaging appearance. Radiol Case Rep.

[6] Zhang C, Jia C-J, Xu C, Sheng Q-J, Dou X-G, Ding Y (2020). Undifferentiated embryonal sarcoma of the liver: clinical characteristics and outcomes. World J Clin Cases.

[7] Stocker JT, Ishak KG (1978). Undifferentiated (embryonal) sarcoma of the liver: report of 31 cases. Cancer.

[8] Baron PW, Majlessipour F, Bedros AA, Zuppan CW, Ben-Youssef R, Yanni G (2007). Undifferentiated embryonal sarcoma of the liver successfully treated with chemotherapy and liver resection. J Gastrointest Surg.

[9] Kamrani K, Patel A, Guerrieri C, Bethel CAI, Phatak T (2019). Undifferentiated embryonal sarcoma of the liver mimicking venolymphatic malformation. Radiol Case Rep.

[10] Plumblee L, Grey H, Hudspeth M, Nadig S (2019). Undifferentiated embryonal sarcoma and the role of liver transplantation. J Pediatr Surg Case Rep.

[11] Shi Y, Rojas Y, Zhang W, Beierle EA, Doski JJ, Goldfarb M (2017). Characteristics and outcomes in children with undifferentiated embryonal sarcoma of the liver: a report from the National Cancer Database. Pediatr Blood Cancer.

[12] Khan ZH, Ilyas K, Khan HH, Ghazanfar H, Hussain Q, Inayat F (2017). Unresectable undifferentiated embryonal sarcoma of the liver in an adult male treated with chemotherapy and orthotopic liver transplantation. Cureus.

[13] Lightfoot N, Nikfarjam M (2012). Embryonal sarcoma of the liver in an adult patient. Case Rep Surg.

[14] Hung TY, Lu D, Liu MC (2007). Undifferentiated (embryonal) sarcoma of the liver complicated with rupture in a child. J Pediatr Hematol Oncol.

[15] Pandit N, Jaiswal LS, Shrestha V, Awale L, Adhikary S (2019). Undifferentiated embryonal sarcoma of liver in an adult with spontaneous rupture and tumour thrombus in the right atrium. ANZ J Surg.

[16] Hu H-M, Zhang W-L, Li J, Wen Y, Li F, Zhi T (2019). Report of seven children with undifferentiated embryonal sarcoma of the liver. Chin Med J..

[17] Shi M, Xu H, Sangster GP, Gu X (2018). Pulmonary metastases from an undifferentiated embryonal sarcoma of the liver: a case report and review. Case Rep Oncol Med.

[18] Stocker JT (2001). Hepatic tumors in children. Clin Liver Dis.

[19] Hiyama E (2014). Pediatric hepatoblastoma: diagnosis and treatment. Transl Pediatr.

[20] Aronson DC, Meyers RL (2016). Malignant tumors of the liver in children. Semin Pediatr Surg.

[21] Kiruthiga KG, Ramakrishna B, Saha S, Sen S (2018). Histological and immunohistochemical study of hepatoblastoma: correlation with tumour behaviour and survival. J Gastrointest Oncol.

[22] Stringer MD, Alizai NK (2005). Mesenchymal hamartoma of the liver: a systematic review. J Pediatr Surg.

[23] Kinariwala DJ, Wang AY, Melmer PD, McCullough WP (2017). Embryonal rhabdomyosarcoma of the biliary tree: a rare cause of obstructive jaundice in children which can mimic choledochal cysts. Indian J Radiol Imaging.

[24] Silva Cunha JL, De Assis Almeida Lima-Júnior F, Gonçalves Júnior WD, De Santana Santos T, Da Silveira EJD, De Sousa SF, et al. Embryonal Rhabdomyosarcoma (Botryoid subtype) affecting the Buccal Mucosa. Head Neck Pathol. 2019;13(4):671–6. 10.1007/s12105-018-0957-8 PMC6854166 30094776

[25] Pinamonti M, Vittone F, Ghiglione F, Borasi A, Silvestri S, Coverlizza S (2018). Unexpected liver embryonal sarcoma in the adult: diagnosis and treatment. Case Rep Surg.

